# A New Probabilistic Ellipse Imaging Method Based on Adaptive Signal Truncation for Ultrasonic Guided Wave Defect Localization on Pressure Vessels

**DOI:** 10.3390/s22041540

**Published:** 2022-02-17

**Authors:** Qinfei Li, Zhi Luo, Gangyi Hu, Shaoping Zhou

**Affiliations:** School of Mechanical and Power Engineering, East China University of Science and Technology, Shanghai 200237, China; liqinfei@mail.ecust.edu.cn (Q.L.); zhiluo@ecust.edu.cn (Z.L.); gy_hu0202@163.com (G.H.)

**Keywords:** pressure vessel, ultrasonic guided wave, defect location, signal analysis, probabilistic elliptic algorithm, adaptive signal truncation

## Abstract

Pressure vessels are prone to defects due to environmental conditions, which may cause serious safety hazards to industrial production. The probabilistic ellipse imaging method, based on ultrasonic guided wave, is a common method for locating defects on plate-like structures. In this paper, the research showed that the accuracy of the traditional probabilistic ellipse imaging method was severely affected by the truncation length of the signal. In order to improve the defect location accuracy of the probabilistic elliptic imaging algorithm, an adaptive signal truncation method based on signal difference analysis was proposed, and a novel probabilistic elliptic imaging method was developed. Firstly, the relationship model between the signal difference coefficient (SDC) and the distance coefficient was constructed. Through this model, the distance coefficient of each group signal can be calculated, so that the adaptive truncation length for each group of signals can be determined and the truncated signals used for defect imaging. Secondly, in order to improve the robustness of the new imaging method, the relationship between the defect location accuracy and SDC thresholds were investigated and the optimal threshold was determined. The experimental results showed that the probabilistic ellipse imaging algorithm, based on the new adaptive signal truncation method, can effectively locate a single defect on a pressure vessel.

## 1. Introduction

Pressure vessels are devices that can hold gas or liquid under a certain pressure, which are widely used in industry production, civil industries, etc. Under the influence of actual working conditions and environment, pressure vessels are prone to cracks, corrosion, holes, and other defects, which may cause safety hazards to industrial production [1]. Therefore, in order to improve the working safety of pressure vessels, it is necessary to detect and locate the defects on pressure vessels. Ultrasonic guided wave testing technology is one of the more popular non-destructive testing technologies used in recent years. Compared with traditional non-destructive testing technologies, ultrasonic guided wave testing is widely used with the advantages of simpler detection equipment, larger detection range, faster detection speed, and online monitoring. Ultrasonic guided wave testing has also been applied in the structural health monitoring of pressure vessels and has achieved certain results [2,3,4]. At present, the research of ultrasonic guided wave defect detection mainly focuses on plate and pipe structure, with few studies reporting on pressure vessels. In terms of the propagation characteristics of guided waves, Li et al. [5] studied the dispersion characteristics of different guided wave modes in pressure vessels, and optimized the excitation center frequency and waveform parameters of guided waves according to the simulation and experimental results. Yang et al. [6] proposed an elliptic positioning algorithm based on coordinate transformation, based on the propagation characteristics of guided waves in hollow spheres and cylinders. Zhai et al. [7] studied the direct wave difference values of different distance coefficients in pressure vessels, and Chen et al. [8] proposed a fuzzy C-means clustering algorithm based on direct wave. Although some scholars have done some work around the defect detection of pressure vessels based on guided wave technology, the imaging algorithm and positioning accuracy are still worthy of further research.

The probabilistic ellipse algorithm is a commonly used imaging algorithm based on ultrasonic guided wave to detect defects in plate-like structures. For defect detection and localization in flat-panel components, the probabilistic ellipse imaging algorithm has relatively mature applications. Many scholars have studied the improvement and application of the probabilistic ellipse algorithm. Liu et al. [9] used the probabilistic elliptic positioning algorithm to perform imaging of artificial defects on board members, and the experimental results showed that the direction and shape of the artificial defects could be effectively identified. Tua et al. [10] estimated defect-reflected wave time-of-flight (ToF) through the energy spectrum of the Hilbert–Huang transform, and used the probabilistic elliptic positioning algorithm to locate defects in plates. This provides a basis for the signal difference analysis of ultrasonic guided wave. Li et al. [11] combined wavelet transform and the probabilistic elliptic imaging algorithm to realize the location of artificial defects in a curved, spherical, thin shell structure. Chen et al. [12] proposed a signal screening method based on the distance coefficient on the basis of the traditional probabilistic elliptic imaging algorithm, and the experiments showed that the specific data filtered by the distance coefficient could improve the defect positioning accuracy of plate structures. These studies have improved the imaging algorithm through signal analysis and signal preprocessing. This idea inspired the following research in this paper. Hu et al. [13] studied the propagation characteristics of guided waves in 30 CrMo steel bent plates with different radii and depths using experimental and numerical methods, and used the probabilistic elliptic imaging algorithm to locate defects in bent plates. In these studies, the probabilistic elliptic imaging algorithm was widely applied in the defect detection of pressure vessels based on guided waves, but the feasibility of the probabilistic elliptic imaging algorithm for different arrays has not been discussed. In particular, the signal truncation strategy, which plays a decisive role in imaging accuracy, has also not been discussed. There are few studies on the imaging accuracy of the probabilistic elliptic imaging algorithm applied to the defect detection of pressure vessels. Therefore, the probabilistic ellipse imaging algorithm for defect detection in pressure vessels is worth studying.

The feasibility of the traditional probabilistic elliptic imaging algorithm for defect detection and location in pressure vessels will be studied in this paper. In order to improve the accuracy of the imaging positioning, the factors affecting the imaging accuracy should be discussed. In this study, a new adaptive signal truncation method based on signal difference analysis is proposed. The structure of this paper is as follows. The factors affecting the imaging accuracy of the traditional probabilistic elliptic imaging algorithm for defect detection in pressure vessels are discussed in Section 2. In Section 2, the concepts of the signal difference coefficient (SDC) and the distance coefficient are briefly introduced, and a new idea of signal truncation based on signal difference analysis is constructed. The experimental objects and parameters of the experimental operations are described in detail in Section 3. In Section 4, the relationship model between the SDC and the distance coefficient is constructed based on experimental data, and the new adaptive signal truncation method is verified. Then, the imaging accuracy of the probabilistic ellipse imaging algorithm based on the new method is discussed and further improved. Finally, the conclusion is given in Section 5.

## 2. Theory

### 2.1. Traditional Probabilistic Elliptic Imaging Algorithm and Signal Processing

**Figure 1:**
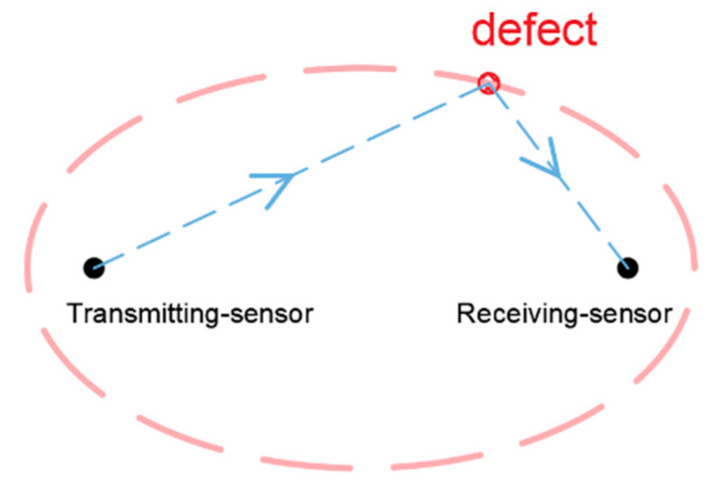
Ellipse trajectory of defect location.

The probabilistic ellipse imaging algorithm is an algorithm developed based on the ellipse imaging algorithm. Both are localization algorithms based on the time-of-flight of scattered waves. By calculating the difference in signal between the healthy signal and the damaged signal, the peak wave packet of the defect scattered wave can be obtained. The arrival time *t*_1_ of the scattered wave is generally taken as the time when the peak of the scattered wave appears. *t*_1_ represents the time taken for the guided wave to propagate from the excitation source to the defect to the receive sensor. Thus, the sum of the distances of the defect to the two sensors can be calculated. According to the sum of the distances from the defect to the two sensors, the possible trace of the defect can be obtained, like an ellipse, as shown in Figure 1. The location of the defect can be determined from the intersection of the elliptical trajectories of multiple sensor pairs. Based on the ellipse imaging algorithm, the probabilistic ellipse imaging algorithm combines the probability functions, and then the entire area of the detected object is meshed. The distance from each cell to the elliptical trajectory is calculated, and a probability function is used to express the probability of the defect location in that cell. After all groups of signals are processed, the position of the maximum probability value in the imaging result is considered as the position of the defect.

**Figure 2:**
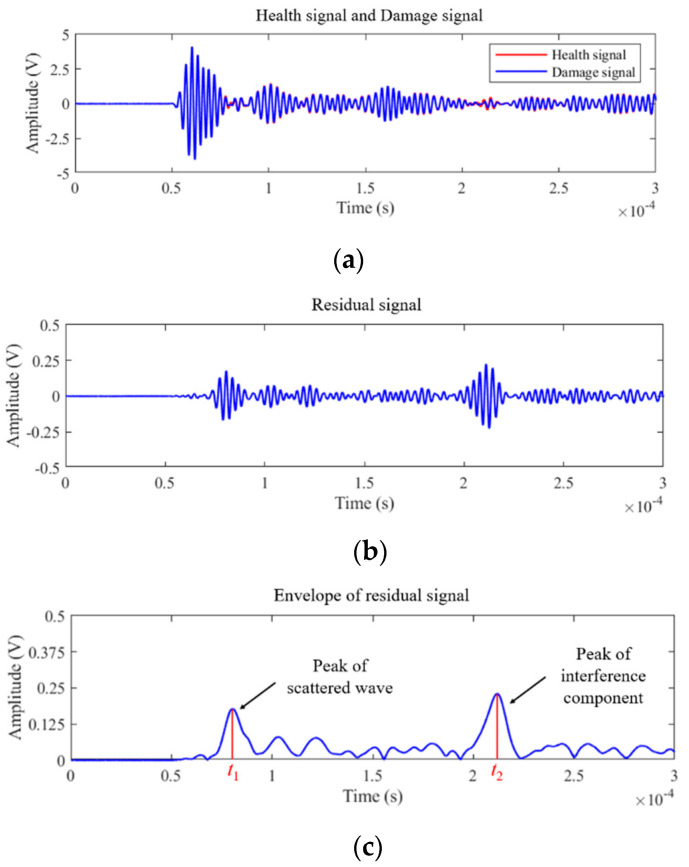
Signal processing of the traditional elliptic imaging method: (**a**) healthy signal and damaged signal; (**b**) residual signal obtained by baseline subtraction. (**c**) Envelope of residual signal.

In traditional probabilistic elliptic imaging, the guided wave signal is analyzed by using baseline subtraction to extract the scattered wave packet. Firstly, the ultrasonic guided wave signal acquired by the experiment is preliminarily filtered and denoised. Then, the damaged signal is subtracted from the healthy signal to obtain the residual signal containing the defect scattered wave. Finally, the envelope of the residual signal is obtained by Hilbert transformation. A case of signal processing from the defect detection experiment on pressure vessel is shown in Figure 2.

By analyzing the envelope signal (Figure 2c), it can be found that in addition to the scattered signal caused by the defect, there are also interference components. These interference components may be caused by factors such as multiple reflected wave and electromagnetic noise. In Figure 2c, it can be seen that the amplitude of the interference component peak is even greater than the scattered wave. In this case, the arrival time of the interference component packet (*t*_2_), rather than the arrival time of the scattered wave packet (*t*_1_), is easy to be mistaken for the index representing the defect. In order to avoid the influence of these interference signals on the imaging accuracy, the signal should be truncated after the abovementioned signal processing steps.

### 2.2. Signal Difference Coefficient and Signal Truncation

In order to avoid the influence of unimportant signals on defect detection, the difference analysis of the signal is carried out. The signal difference coefficient (SDC) was first used in rapid algorithms in ultrasonic guided wave imaging technology to evaluate the correlation between a healthy signal and a damaged signal [11], and the equation is:

(1) $$\mathrm{SDC}=1-\frac{C_{XY}}{\sigma_{X}\sigma_{Y}}$$

where $C_{XY}$ is the covariance of signal *X* and *Y*, $\sigma_{X}$ and $\sigma_{Y}$ are the variance of signal *X* and signal *Y*.

(2) $$C_{XY}=\sum_{k=1}^{K}\left(X_{k}-\mu_{X})(Y_{k}-\mu_{Y}\right)$$

(3) $$\sigma_{X}\sigma_{Y}=\sqrt{\sum_{k=1}^{K}{\left(X_{k}-\mu_{X}\right)}^{2}}\sqrt{\sum_{k=1}^{K}{\left(Y_{k}-\mu_{Y}\right)}^{2}}$$

where μ is the mean value of the signal amplitude and K is the signal length.

**Figure 3:**
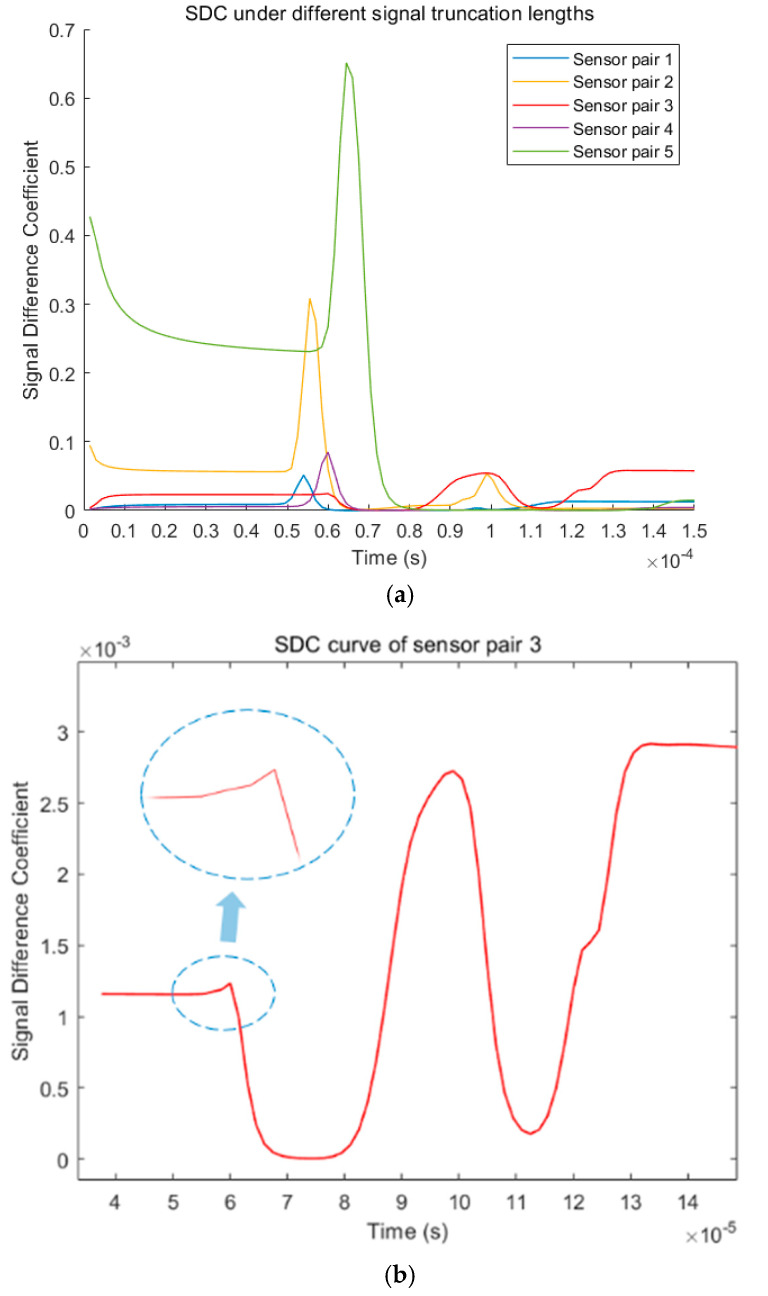
Signal difference analysis of healthy signal and damaged signal under different truncated length: (**a**) SDC curves of different sensor pairs; (**b**) SDC curves of sensor pair 3.

In the traditional probabilistic ellipse imaging algorithm, the signals of all groups are usually truncated at a fixed length of time. The arrival time of boundary reflection/diffraction wave (*t_s_*) is usually used as the fixed length. Theoretically, the amplitude of the scattered wave packets caused by defect reflection in the truncated signal should be more prominent. However, in practice, some interference components may be larger than the residual signals, like the case in Figure 2c. Therefore, different truncation lengths should be discussed. Rather than residual signal analysis, signal difference analysis is conducted. Figure 3 shows the signal difference analysis between the healthy signal and the damaged signal between the sensor pairs of different paths after truncation. Before *t_s_*, 100 equidistant signal interception is performed. The time of each curve’s peak in Figure 3 corresponds to the arrival time of each group of damaged reflection wave signals (*t*_1_). In the curves of sensor pairs 1, 2, 4, and 5, the damaged reflection waves obviously correspond with the curve’s peak. Figure 3b shows the SDC curve of sensor pair 3. According to the experimental parameters, the SDC peak value of sensor pair 3 should appear where it is circled in Figure 3b. However, with the extension of the truncation time, a peak that is not related to the damaged reflection signal was shown in the curve.

From Figure 3a, the fixed truncation length used in the whole group is not suitable for the signal of sensor pair 3. If each group of signals is truncated at a fixed length, some interference components that are more prominent than the scattered wave packages from defects may be included in the truncated signals. The actual ellipse track will be missed due to these truncated signals in the defect location. When the processed signal contains multiple interference components, the pixel value at the defect position in the imaging result will be sharply reduced, which will eventually lead to the failure of defect localization. Therefore, in order to accurately locate defects, appropriate truncated lengths must be selected for each group of signals to overcome the influence of interference components.

### 2.3. Signal Difference Coefficient and Signal Truncation

In actual ultrasonic guided wave defect detection and positioning experiments, the large deviation of the imaging results is caused by the inaccurate truncated length. Due to the uncertainty of the defect location information and the difference in the relative position of the sensor pairs, the optimal truncated length is inconsistent. In order to obtain more accurate imaging results, the relationship between the optimal truncated length and the distance coefficient of the signals will be discussed in this section.

Figure 3a shows that the peak values of different curves have different amplitudes and delay times. According to Chen’s studies [12], the peak value of the SDC varies with the distance between defects and the path of a transmitter/receiver pair. Figure 3 shows that the damaged reflected signal in each group of signals will cause SDC mutations, and this mutation can be observed even in sensor pair 3. However, the severity of the mutation is different in these signals. In order to adaptively determine the signal’s truncated length, it is necessary to analyze the relationship between the arrival time of the scattered waves and the peak value of the SDC mutation. The arrival time of the damaged reflection wave is related to the defect position and the distance of the sensor pair. Therefore, for the convenience of description, the concept of distance coefficient $c_{d}$ is defined, and it is defined as in Equation (4):

(4) $$c_{d}=\frac{d_{TD}+d_{RD}}{d_{TR}}=\frac{d_{TRD}}{d_{TR}}$$

where $d_{TR}$ is the distance from the excitation sensor to the receiving sensor; $d_{TD}$ is the distance from the excitation sensor to the defect; $d_{RD}$ is the distance from the defect to the receiving sensor; and $d_{TRD}$ is the sum of the distances from the excitation sensor to the defect and from the defect to the receiving sensor. Then, the ideal truncation time *t*_1_ can be expressed by Equation (5) [12]:

(5) $$t_{1}=c_{d}d_{TR}/v_{g}$$

where $v_{g}$ is the group velocity of the S0 mode of the ultrasonic guided wave.

If the signal truncated time is set to *t*_1_, the wave package from the defect is included in the truncated signal and the interference components are excluded. However, the defect location is unknown in the actual detection, and the distance coefficient of each sensor pair cannot be determined directly. Although the arrival time of the scattered waves cannot be obtained directly, Equation (5) provides a method to estimate the *t*_1_ of the scattered waves using a distance coefficient. Relevant studies have shown that with the increase of the distance between a defect and sensor pairs, the difference between the healthy signal and damaged signal will decrease [14,15]. According to this characteristic, the distance coefficient can be judged by the difference between the healthy signal and damaged signal, and then the *t*_1_ of the scattered wave can be estimated. In order to determine the optimal truncated length, the relationship between the peak value of the SDC mutation and the distance coefficient should be studied. For the convenience of presentation, the equational relationship between the SDC and the distance coefficient is expressed as the exponential equation form of Fourier series:

(6) $$S(c_{d})=F_{0}+\sum_{n=1}^{n}\left(F_{n}\cdot e^{jn\omega c_{d}}+F_{-n}\cdot e^{-jn\omega c_{d}}\right)$$

where n is the number of series to expand, ω is the angular frequency, $F_{0}$ is the constant term to be solved, and $F_{n}$ and $F_{-n}$ are the coefficients of the two terms of the nth level in the expansion.

Obviously, the relationship between SDC and the distance coefficient monotonically decreases, so the above equation is simplified to Equation (7). Through the experimental data in Section 3 and the discussion in Section 4, the specific parameters in Equation (7) will be determined and the accuracy of the formula will be verified.

(7) $$S(c_{d})=a_{0}+b_{0}\cdot e^{f(c_{d})}$$

where $a_{0}$ is the constant term to be solved, and $b_{0}$ is the coefficient of the exponential term to be solved.

**Figure 4:**
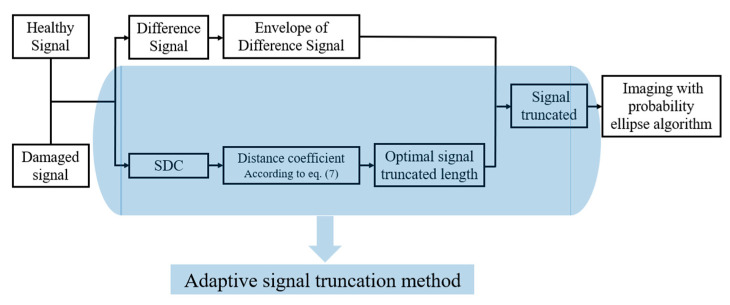
Imaging process of the probabilistic elliptical algorithm based on the adaptive signal truncation method.

In order to solve the relationship equation between the SDC and the distance coefficient, the signals of each sensor pair need to be tested at different defect positions. The experimental setup is described in detail in Section 3. The technical route of the new signal difference analysis method is shown in Figure 4. Firstly, the SDC mutation of the healthy signal and the damaged signal will be calculated according to Equation (1). Then, according to Equation (7), the distance coefficient can be estimated. Finally, the optimal truncated lengths for different group signals can be determined.

## 3. Experiment

### 3.1. Experiment Platform

**Figure 5:**
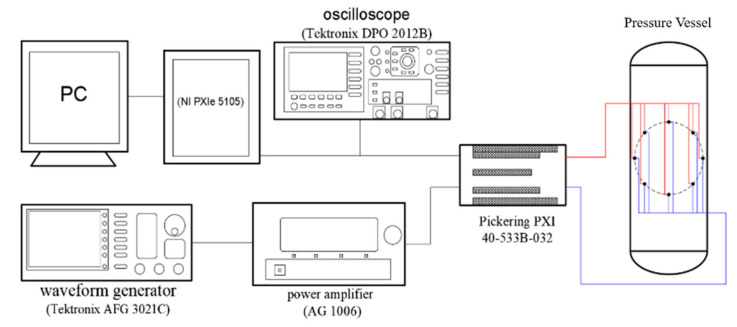
Experimental system.

In this study, the whole experimental platform of multi-channel ultrasonic guided wave detection and the connection of the system are shown in Figure 5. The experimental detection steps are as follows. Firstly, five-cycle sinusoidal wave signals modulated by the Hanning window are generated by a waveform generator (Tektronix AFG 3021C, Tektronix，Beaverton, OR, USA), and then the signals are amplified using a power amplifier (AG 1006, TC Power Conversion, Rochester, NY, USA) and transmitted to one piezoelectric sensor. The piezoelectric sensor converts the electrical signal into a vibration signal through the inverse piezoelectric effect, and then the ultrasonic guided wave signal is excited in the pressure vessel. After the ultrasonic guided wave vibration signal propagates in the pressure vessel, the corresponding vibration signal is received by the piezoelectric sensors in other positions. The guided wave vibration signals are then converted into electrical signals by the piezoelectric effect and displayed on an oscilloscope (Tektronix DPO 2012B, Tektronix，Beaverton, OR, USA), and finally, data are automatically collected and stored in the computer through the data acquisition system (NI PXIe 5105, Pickering PXI 40-533B-032, Apex Waves, Cary, NC, USA).

### 3.2. Experimental Parameters

**Figure 6:**
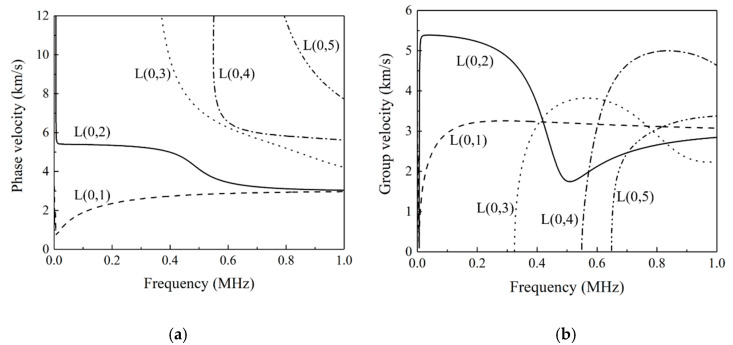
Dispersion curves in 5 mm thick pressure vessel: (**a**) dispersion curve of phase velocity; (**b**) dispersion curve of group velocity.

**Figure 7:**
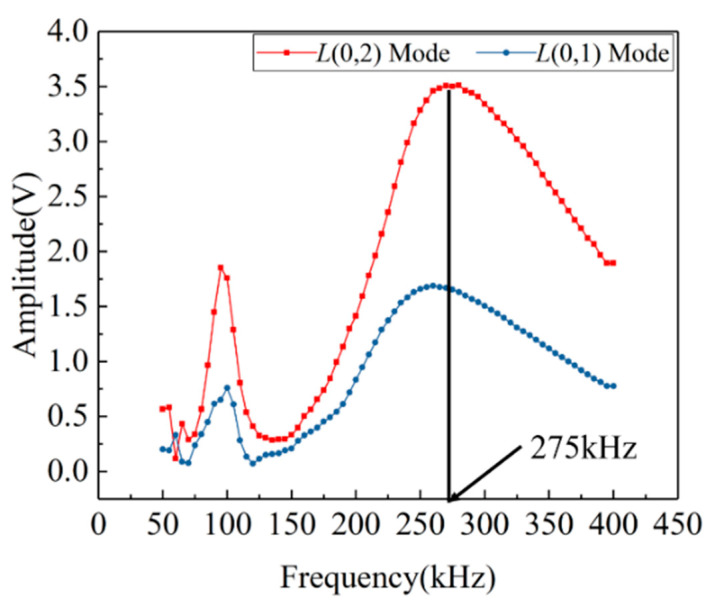
Amplitude–frequency characteristics of L (0,1) and L (0,2) modes.

The size and material composition of the pressure vessel in the experiment are shown in Table 1. The size of the ceramic piezoelectric wafers used to excite and receive the ultrasonic guided waves are 10 mm (diameter) × 1 mm (thickness). The signal sampling frequency involved in the experiment in this paper is 50 MHz, the trigger voltage is set at 200 mV, and the single sampling time is 0.5 ms. According to the dispersion curve and amplitude–frequency characteristic (Figure 6 and Figure 7), the L (0,2) mode guided wave signal under 275 kHz is selected as excited signal for the defect detection experiment.

**Figure 8:**
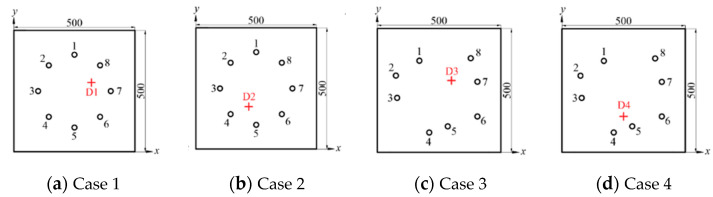
Position of sensors and defects. (“O” represents the positions of the sensors and “+” represents the actual positions of the defects.)

Defects are simulated by bonding a steel ingot (diameter 10 mm) to the surface of the pressure vessel. To visually represent the relative positions of sensors and defects in the four groups, the images of the curved cylinder are converted into 2D flat images, and then the positions of the sensors and defects are quantitatively described on the plane. In order to avoid errors, four groups of experiments with different defect locations and arrays are set up. The locations of the sensors and defects are shown in Figure 8. The coordinates of the sensors and defects in each array are shown in Table 2.

## 4. Results and Discussion

### 4.1. Adaptive Signal Truncation Method Based on Signal Difference Analysis

**Figure 9:**
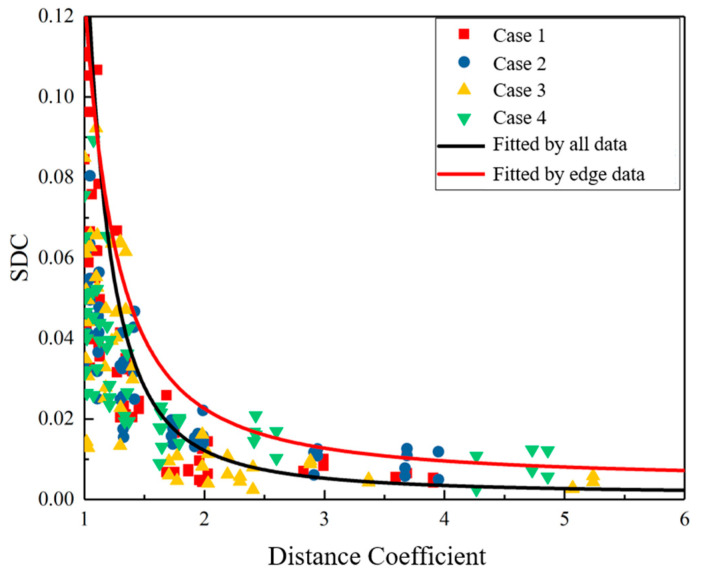
Relationship between distance coefficient and signal difference coefficient.

Through the experiment in Section 3, the data of each case are collected and recorded. The data are shown in Figure 9. It can be seen that the SDC decreases as the distance coefficient increases. Based on these data points, the relationship models of the SDC and the distance coefficient in each case are first established, and then exponential fitting is performed according to the relationship equation of the four cases. The fitting curve is shown as the black curve in the figure. The analytical formula of the curve is as follows:

(8) $$S(c_{d})=0.0013\cdot e^{\frac{4.2922}{c_{d}}}$$

After obtaining the approximate relationship between the SDC and the distance coefficient, the distance coefficient of the sensor pair can be estimated to be *t*_1_. It should be noted that the distance coefficient–SDC curve that fits all data is located in the “middle” of the data. Therefore, when calculating the distance coefficient according to the SDC, the distance coefficient of the “outside” part of the curve is smaller than the actual distance coefficient, resulting in an estimated *t*_1_ that is too small, which may cause the reflected wave packages from defects to be missed. In order to avoid this situation, the edge data points are used to fit the curve, and a new fitting curve is obtained (the red curve in Figure 9). The analytical formula of the new curve is as follows:

(9) $$S(c_{d})=0.0039\cdot e^{\frac{2.4293}{c_{d}}}$$

**Figure 10:**
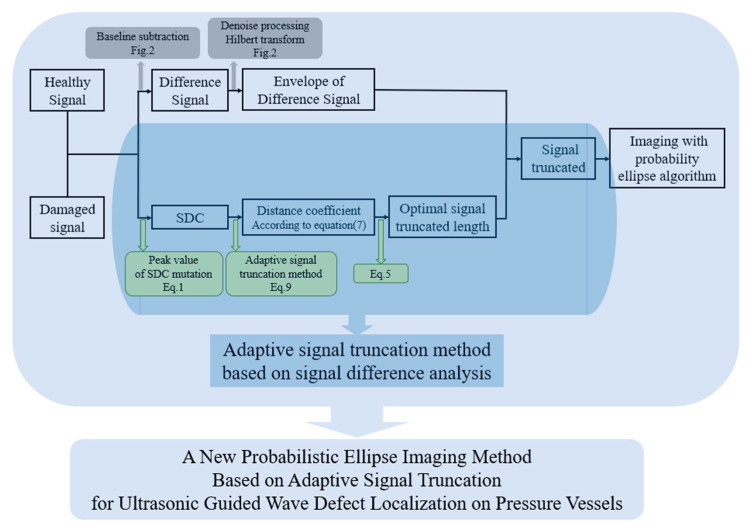
Adaptive signal truncation method based on signal difference analysis.

Thus, the specific parameters of Equation (7) in Section 2.3 are determined through experiments. Therefore, an adaptive signal truncation method based on signal difference analysis is proposed in this study. The technical route of this method, described in Figure 3 in Section 2.3, is supplemented as shown in Figure 10. The imaging effect based on this new signal truncation method will be discussed in the next section.

### 4.2. Analysis of Imaging Effect under New Adaptive Signal Truncation Method

**Figure 11:**
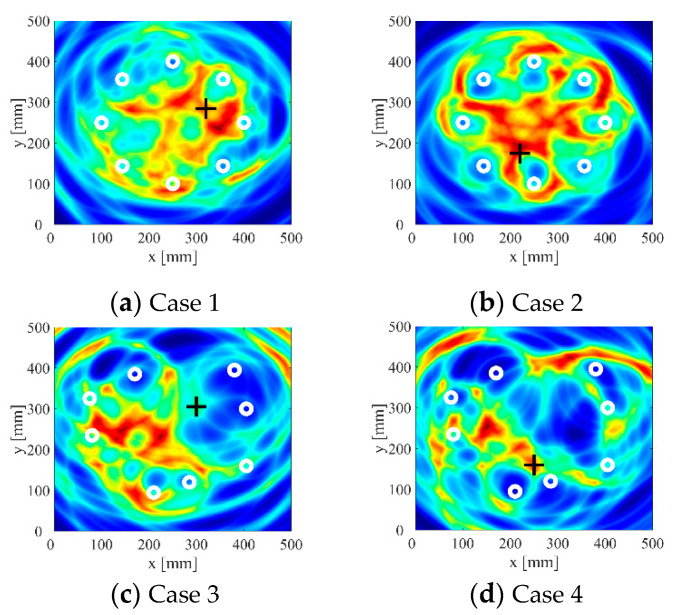
Imaging results of traditional probabilistic ellipse imaging algorithm. (The white circles are the sensor locations, and the black crosses are the actual locations of the defect.)

**Figure 12:**
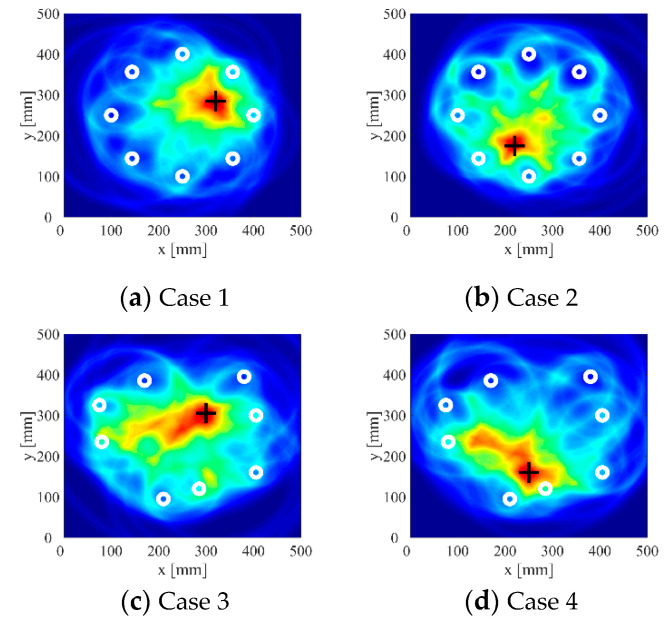
Imaging results of probabilistic ellipse based on adaptive signal truncation method.

In order to explore the effect of the probabilistic ellipse imaging algorithm based on the new adaptive signal truncation method, the improved imaging algorithm is used to image the experimental data. The traditional probabilistic elliptic imaging results are shown in Figure 11. The imaging results are shown in Figure 12. From the imaging results, it can be seen that the image obtained by the improved probabilistic ellipse imaging algorithm can effectively characterize the defect location. The defect location errors of case 1 to case 4 are 5.30 mm, 2.80 mm, 6.25 mm, and 4.51 mm, respectively. In addition, compared with the imaging results in Figure 11, the number and range of artifacts in Figure 12 are significantly reduced. The defect location accuracy is significantly improved. Obviously, the new signal truncation method can be effectively applied to defect detection in pressure vessels.

In Figure 12c,d, the smear outside the defect area can be seen. This phenomenon can be explained as follows: when calculating the distance coefficient according to the SDC–distance coefficient fitting Equation (9), if the SDC is very small, the deviation of the calculated distance coefficient can be large. Moreover, when all signals are directly used for imaging, the program can easily report errors. Therefore, it may be beneficial to set a minimum SDC threshold Q during the imaging process to perform a preliminary screening of signals, and to exclude signals with an SDC less than the threshold Q from the final imaging result.

### 4.3. Threshold Optimization of Adaptive Signal Truncation Method

**Figure 13:**
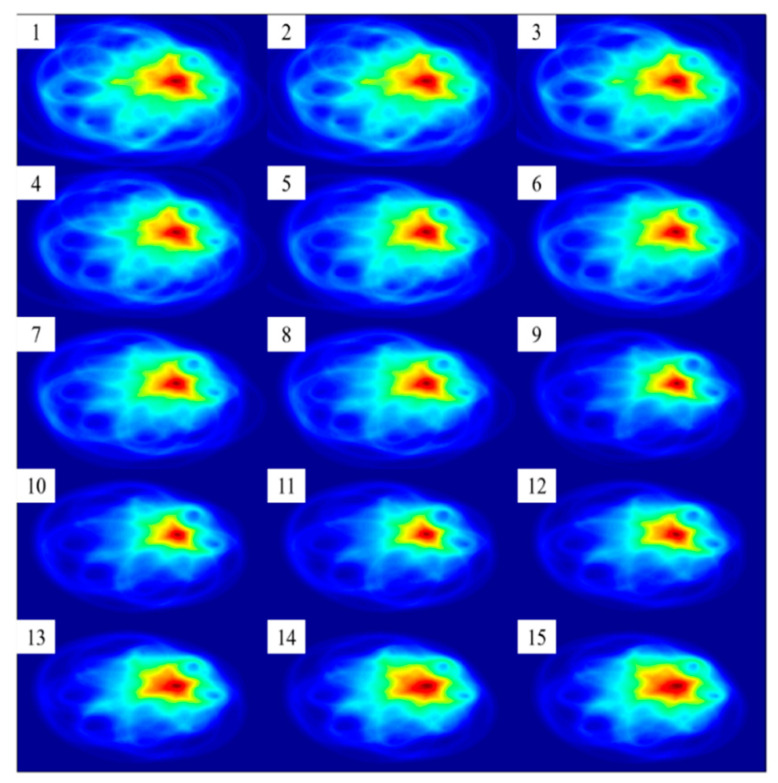
Case 1 imaging results of probabilistic ellipse imaging algorithm at different SDC thresholds.

In order to determine an appropriate threshold Q, the imaging results under different thresholds are compared. In this study, the threshold Q value is set from 0 to 0.03 at 0.002 intervals. The imaging results of Case 1 under 15 different thresholds are shown in Figure 13. Among them, sub-picture 1 corresponds to the imaging result with threshold Q = 0, sub-picture 2 corresponds to the imaging result with threshold Q = 0.002, and so on, and sub-picture 15 corresponds to the imaging result with threshold Q = 0.028. It can be observed that as the threshold Q increases from 0 to about 0.02, the high pixel value area close to the defect position in the imaging result gradually shrinks. That is, the pixel value contrast between the defect position and the non-defect position is improved, and the accuracy of the imaging result is also improved. As the threshold Q increases from 0.02, the high pixel value area of the defect tends to expand, and the imaging quality gradually deteriorates.

In order to quantitatively evaluate the influence of threshold Q on the positioning results, a positioning error is used to quantify the deviation between the defect position in the imaging result and the actual defect position. The aggregation coefficient c [16] is used to quantify the aggregation degree of pixels in the imaging results, that is, the color contrast between the defect area and the non-defect area in the image. The definition of positioning error d is given by Equation (10), and the definition formula of aggregation coefficient c is given by Equation (11):

(10) $$d=\sqrt{{\left(x_{p}-x_{r}\right)}^{2}+{\left(y_{p}-y_{r}\right)}^{2}}$$

(11) $$\hat{c}=\underset{c}{\mathrm{argmin}}\sum {\left(\ln\left(\frac{P(x)}{P(0)}\right)+cx\right)}^{2}$$

where $x_{p}$ and $y_{p}$ represent the horizontal and vertical coordinates of the defect obtained from the imaging results; $x_{r}$ and $y_{r}$ represent the actual horizontal and vertical coordinates of the defect;P(x) represents the pixel value of a discrete point whose distance is *x* from the maximum pixel value point, and in particular, P(0) is the pixel value of the maximum pixel value point.

**Figure 14:**
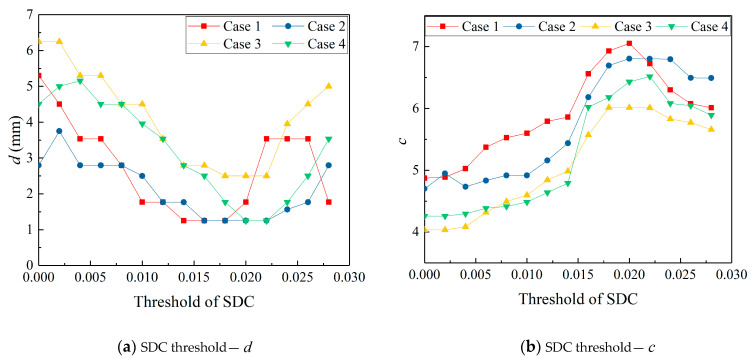
Imaging location deviation and degree of aggregation under different SDC thresholds.

**Figure 15:**
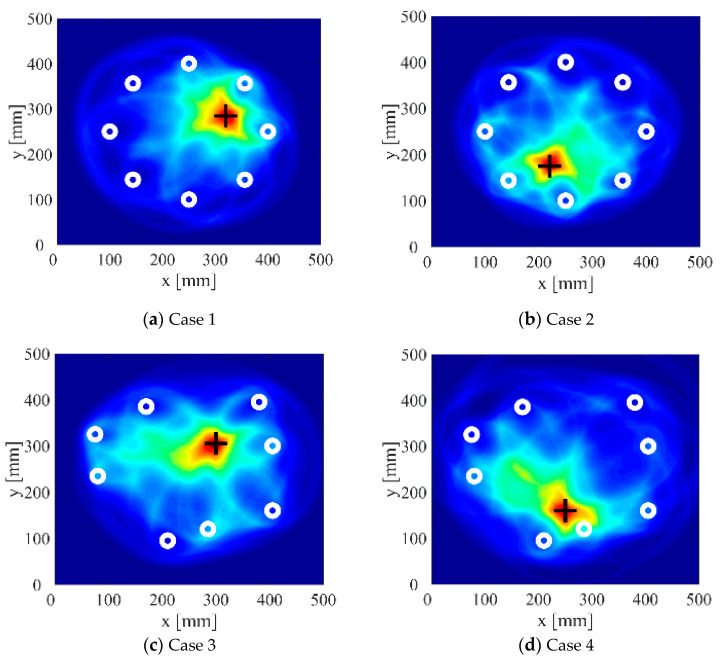
Imaging results with SDC threshold set to 0.02.

According to the definition of clustering coefficient c, when the pixel value contrast increases, the aggregation coefficient also increases. In order to compare the imaging effects under different SDC thresholds *Q*, the defect location errors and aggregation coefficients of different sensors and defects at each threshold *Q* are calculated, as shown in Figure 14a,b. It can be observed that in terms of the positioning error, when the threshold *Q* is in the range of 0–0.02, the positioning error slightly decreases with the increase of the threshold value, but the decrease range is very small; when the threshold *Q* exceeds 0.02, the positioning error begins to increase with the increase of the threshold value. In terms of aggregation coefficient c, when the threshold *Q* is in the range of 0–0.02, the aggregation coefficient c increases with the increase of the threshold value; when the threshold value exceeds 0.02, the aggregation coefficient begins to decrease. Considering the comprehensive influence of the threshold on the positioning error and aggregation coefficient, it can be found that the imaging results with a threshold between 0.018 and 0.022 are better. The imaging results of four cases when the SDC threshold *Q* is set to 0.02 are shown in Figure 15.

In Table 3, the defect location error and aggregation coefficient of the traditional probabilistic ellipse imaging algorithm, the probabilistic ellipse imaging algorithm based on the fixed time signal truncation method, and the new adaptive signal truncation method are listed. (The fixed truncated length is set as 0.10 ms.) It can be seen that the traditional probabilistic ellipse imaging algorithm has the worst localization quality, and its defect location error is much greater than that of the improved algorithm; the fixed time signal truncation method effectively reduces the positioning error of the traditional probabilistic ellipse imaging algorithm, but the improvement of the aggregation coefficient of the positioning result is limited; the results show that the new image algorithm based on the adaptive signal truncation method has the best positioning quality when the threshold set to 0.02, and the positioning accuracy and aggregation coefficient of the positioning results are significantly improved. The minimum positioning error is 1.25 mm, and the maximum aggregation coefficient is 14.10.

It can be seen intuitively from Table 3 and Figure 15 that the signal truncation method based on signal difference analysis proposed in this study can effectively improve the imaging accuracy of the probabilistic ellipse imaging algorithm for single defect detection in pressure vessels.

## 5. Conclusions

The feasibility of the traditional probabilistic ellipse imaging algorithm for defect detection in pressure vessels were theoretically and experimentally studied in this paper. The experimental results show that the imaging results are affected by the signal truncation length. There are large deviations in the imaging results based on the fixed-length truncated signal. On this basis, in view of the confusion of imaging results, the relationship between signal truncation length and imaging accuracy was discussed. In order to improve the positioning accuracy of the probabilistic ellipse imaging algorithm applied to the defect detection on the pressure vessel, experiments, theoretical analysis, and discussion have been conducted in this paper. The main conclusions are as follows:

(i). In this study, it was found that the positioning accuracy of the probabilistic ellipse imaging algorithm is affected by the signal truncation length. By analyzing the healthy signal and the damaged signal, it was found that the truncation length can be determined according to the SDC and the distance coefficient. Then, the functional relationship between the SDC and the distance coefficient was constructed through experiments in this study.
(ii). In this paper, an adaptive signal truncation method based on signal difference analysis is proposed, and based on this, a new probabilistic ellipse imaging method was developed in this study for ultrasonic guided wave defect localization on pressure vessels. The new probabilistic ellipse imaging method can effectively improve the accuracy in detecting and positioning defects on pressure vessels.
(iii). Further study is recommended to analyze the effect of different thresholds of the SDC on the imaging results. The location error and aggregation coefficient are useful for measuring the location accuracy of different imaging methods. The experimental results show that when the SDC threshold is 0.02, the defect location accuracy can be further improved.

## Figures and Tables

**Table 1:** Parameter of pressure vessel.

Material	Outside Diameter (mm)	Inside Diameter (mm)	Height (mm)	Density (kg/mm^3^)	MOE (GPa)	Poisson’s Ratio
30 CrMo	350	340	1000	7850	210	0.30

**Table 2:** Coordinates of sensors and defects.

Sensor/Defect Number	Circular Array	Irregular Array
S1	(250,400)	(170,385)
S2	(144,356)	(75,325)
S3	(100,250)	(80,235)
S4	(144,144)	(210,95)
S5	(250,100)	(285,120)
S6	(356,144)	(405,160)
S7	(400,250)	(405,300)
S8	(356,356)	(380,395)
D1	(320,275)	-
D2	(220,175)	-
D3	-	(300,305)
D4	-	(250,160)

**Table 3:** Deviations and aggregation coefficients of localization results of different probabilistic ellipse imaging algorithms.

Imaging Algorithms		Case 1	Case 2	Case 3	Case 4
(1) Traditional probabilistic ellipse imaging algorithm without signal truncation	Positioning error *d*	48.54	17.15	106.09	137.39
	Aggregation coefficient *c*	0.89	0.89	0.82	0.92
(2) Probabilistic ellipse imaging algorithm with fixed signal truncation length	Positioning error *d*	13.51	15.61	22.21	25.61
	Aggregation coefficient *c*	1.74	1.67	1.60	1.71
(3) Probabilistic ellipse imaging algorithm based on adaptive signal truncation method (no threshold)	Positioning error *d*	5.30	2.80	6.25	4.51
	Aggregation coefficient *c*	9.75	9.40	8.07	8.52
(4) Probabilistic ellipse imaging algorithm based on adaptive signal truncation method (set threshold = 0.02)	Positioning error *d*	1.77	1.25	2.50	1.25
	Aggregation coefficient *c*	14.10	13.61	12.03	12.86

## Data Availability

Not applicable.
